# Investigating low uptake of tuberculin skin testing when integrated with active case-finding for TB

**DOI:** 10.5588/ijtldopen.24.0667

**Published:** 2025-03-12

**Authors:** T.S. Johnson, A. Nalutaaya, P. Biché, K. Ndyabayunga, R. Okura, I. Mugabi, D. Nantale, V. Nakiiza, P.J. Kitonsa, E.A. Kendall, A. Katamba, D.W. Dowdy

**Affiliations:** ^1^ Johns Hopkins Bloomberg School of Public Health, Epidemiology, Baltimore, MD, USA;; ^2^ Walimu, Uganda Tuberculosis Implementation Research Consortium. Kampala, Uganda;; ^3^ Johns Hopkins University School of Medicine, Department of Medicine, Baltimore, MD, USA;; ^4^ Makerere University College of Health Sciences, Department of Internal Medicine, Clinical Epidemiology and Biostatics Unit, Kampala, Uganda.

**Keywords:** tuberculosis, TBI, prevention/control program, epidemiology, diagnosis

Dear Editor,

TB preventive treatment (TPT) has high efficacy in preventing TB disease among individuals with TB infection (TBI).^1^ Broader screening and treatment will be necessary to address the substantial global reservoir of individuals with TBI.^2–4^ Scale-up of TPT in high-burden settings has been hampered by limited diagnostic accuracy, poor durability of protection and health system barriers.^1,5^ Integrating TPT with active case finding can empower screening teams to rule out active TB and provide TPT directly.^6^ Combined screening and TPT linkage has shown sustained reductions in TB burden in specific settings and modeling studies, but more data is needed to inform feasibility in varied contexts.^4,5,7,8^

We integrated tuberculin skin testing (TST) for TBI into a pragmatic cluster-randomized trial of active TB case finding in peri-urban Uganda from June 2022 to April 2024. This ongoing trial compares the effectiveness of two location-based approaches (community-based versus facility-based) to TB screening using mobile chest X-ray (CXR) (Clinicaltrials.gov: NCT05285202).^9^ Participants aged ≥15 years received CXR analyzed by computer-aided detection (qXR v3, Qure.ai, India), with sputum Xpert testing offered to those above a qXR score threshold (initially 0.5, subsequently lowered to 0.1 to enhance sensitivity for scientific purposes). Participants with qXR scores below this threshold were eligible for TST; asymptomatic participants with scores above this threshold were also offered TST while awaiting Xpert results. Participants accepting TST placement were advised to return to the study site in 40–96 hours for reading, after which individuals with induration greater than or equal to 10 mm (and negative Xpert result if tested) were referred to nearby routine health facilities for TPT evaluation. Our primary outcome for this analysis was TST uptake, measured as the proportion of eligible participants completing TST placement and, separately, the proportion returning for TST reading. All consenting participants completed an intake survey on demographics, HIV status, TB symptoms, prior TB, and contact history. A random subset completed a detailed questionnaire on occupation, healthcare-seeking behavior, costs incurred for seeking care, travel times to the screening site and to the nearest health facility, and a rating of overall general health. We performed multiple logistic regression to estimate individual-level associations with successful receipt of TST placement and, separately, return for results after adjusting for variables known a-priori to be risk factors for TB and/or to reasonably influence the ability to participate in TST screening. Finally, we administered a supplemental survey to a consecutive sample of participants accepting TST placement and an equally sized random sample of participants declining TST placement. In the supplemental survey, designed using the theoretical domains framework (TDF),^10^ participants were asked to select and rank up to three challenges to returning for TST reading from a pre-specified list.

Of 33,213 eligible participants, 3,920 (12%) received TST placement, of whom 2,173 (55%) returned for TST reading; 24% traveled 30–60 minutes to the screening site, and an additional 13% of travel exceeded 1 hour. The odds of receiving TST placement were lower for participants who traveled longer to get to the screening site (adjusted odds ratio [aOR] = 0.88, 95% confidence interval [CI] 0.78–0.99, for each 10-minute increase in travel time). Conversely, the odds of TST placement were higher among the 281 (1.0%) participants with a known TB contact (aOR = 1.18, 95% CI 1.09–1.28) and the 910 (3.3%) participants with a previous TB test (aOR = 1.93, 95% CI 1.50–2.45). Participants reporting symptoms in the past 30 days (*n* = 21,464, 65%) were more likely to receive TST placement (aOR = 1.39, 95% CI 1.27–1.54) and to return for TST reading (aOR = 1.35, 95% CI 1.12–1.64). Among 491 participants completing the supplemental survey about barriers to completing TST, 275 received TST placement (56%), and 131 (27%) returned for TST reading. The Figure summarizes the percentage of participants rating each listed barrier as their top perceived challenge to returning. The most prevalent perceived challenge was being busy with other priorities (*n* = 232/491, 47%), while participants who declined TST placement were most likely to cite the ‘financial cost of returning’ as their top challenge (n = 86/216, 40%).

**Figure.**
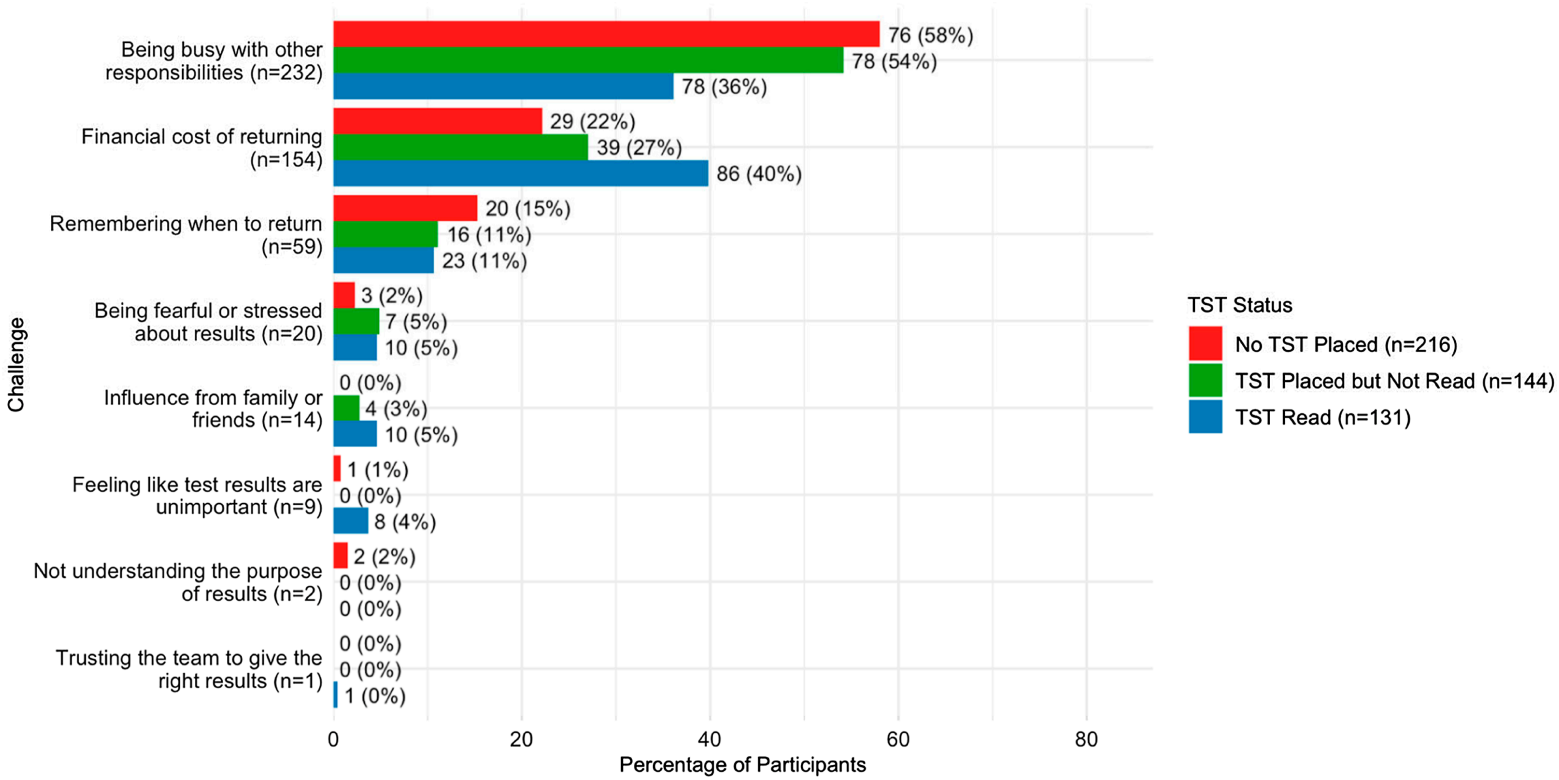
Key barriers to follow-up visits for TST readings during active TB case-finding in Uganda. Bars show the number of participants in community-based or facility-based active case-finding campaigns (total *n* = 491) who selected each challenge listed as the most important barrier to having a TST read. Blue bars show participants (*n* = 216) who declined TST placement, green bars participants (*n* = 144) who had TST placed but did not return for reading, and red bars participants (*n* = 131) who successfully returned for TST reading despite the challenges listed. TST = tuberculin skin test.

In attempting to integrate TST into a CXR-based screening program for the general population of a high-burden setting, we completed TST in only 12% of eligible individuals. Several difficulties limited successful TST completion and TPT linkage. Financial constraints, including the cost and time of transport for return visits, were a major barrier to the implementation of TST screening and TPT linkage. Even when participants were informed that TPT drugs would be provided free of charge, participants declining TST placement were most likely to cite financial barriers, and longer travel time to the screening site was associated with lower odds of successful return for TST reading. Individual-level motivational factors also appeared to influence participation in TST screening, with TB contacts, previous TB testing, and the presence of symptoms all associated with greater odds of TST placement and/or reading. Finally, intervention components inherent to the community-based nature of the campaign posed logistical challenges, including frequent relocation of screening sites, misalignment of screening schedules with TST reading windows, weather-related interruptions and tuberculin shortages.

Although this study leveraged a large case-finding study that likely reached a representative participant population, it had limitations. The applicability of our conclusions is limited to contexts that might implement similar implementation strategies. The location of screening and the ease with which community members would be able to return for results is expected to substantially impact TST screening uptake. There may also be trade-offs between offering TST in a convenient location for participants versus a location that facilitates easier linkage to TPT if positive, an outcome we did not quantify in this analysis. To limit the time burden on participants, we used a short survey that, although informed by grounded theory, had not been validated to assess barriers to TST/TPT. Results from this survey may, therefore, not be fully generalizable to other contexts but could inform future qualitative studies that evaluate these barriers in greater depth and identify implementation strategies to overcome them.

Integrating TBI screening with active case finding for TB represents an opportunity to leverage existing program infrastructure to identify individuals with TBI and initiate TPT. However, we observed a low uptake of TST screening steps, driven by several potential barriers. Without additional mechanisms to streamline screening, integration of screening and TBI treatment within large active case-finding campaigns may have limited impact. TB programs seeking to integrate TBI screening and TPT into community screening interventions should invest in efforts to understand potential barriers and implement strategies to overcome these.
